# The stress reduction potential of Bhagavad Gita and Yoga for healthcare workers during the COVID-19 pandemic: A randomized controlled trial

**DOI:** 10.1371/journal.pone.0347320

**Published:** 2026-04-24

**Authors:** Nishant Das, Laalithya Konduru, Simranjeet Singh Dahia, Santwana Sagnika, Gargi Kothari-Speakman, Ajit Kumar Behura

**Affiliations:** 1 Department of Humanities and Social Sciences, Indian Institute of Technology (ISM), Dhanbad, Jharkhand, India; 2 Department of Community Medicine, Sri Jagannath Healthcare and Research Center, Dhanbad, Jharkhand, India; 3 College of Medicine and Public Health, Flinders University, Bedford Park, South Australia, Australia; 4 Harvard T.H. Chan School of Public Health, Harvard Univresity, Boston, Massachusetts, United States of America; 5 Mineral Resources, Commonwealth Scientific and Industrial Research Organisation, Waite Campus, Urrbrae, South Australia, Australia; 6 School of Computer Engineering, Kalinga Institute of Industrial Technology, Bhubaneswar, Odisha, India; 7 Savitri Ghantasala Center for Health Equity, Samanjasa Foundation, Chennai, Tamil Nadu, India; SDM College of Medical Sciences and Hospital, INDIA

## Abstract

**Background:**

Healthcare workers (HCWs) experienced elevated psychological distress during the COVID-19 pandemic. Culturally embedded, non-stigmatizing approaches may improve uptake of mental health support in this population. We aimed to evaluate the impact of Yoga, Bhagavad Gita learning, and their combination (Yoga+Gita) on psychological distress among HCWs.

**Methods:**

We conducted a prospective, single-center, parallel-group randomized controlled trial at a secondary hospital in India. Eligible HCWs (>18 years, without pre-existing diagnosed mental health conditions) were randomized in a 1:1:1:1 ratio to: Yoga (daily 60-min sessions for one week), Gita (daily 60-min teaching of Bhagavad Gita Chapter 2 for one week), Yoga+Gita (30 min Yoga + 30 min Gita daily for one week), or Control (usual care). Psychological distress was assessed using the Generalized Anxiety Disorder-7 (GAD7) scale at baseline (B-GAD7), immediately post-intervention (IPI-GAD7), and 45 days post-intervention (DPI-GAD7). Participants were aware of group assignment, but data analysts were blinded. Group comparisons used Friedman and Kruskal–Wallis tests with Bonferroni-adjusted post-hoc analyses. We hypothesized that (1) all three interventions would reduce GAD-7 scores compared with control, (2) Gita would produce more durable effects than Yoga alone, and (3) the combined Yoga+Gita intervention would yield the greatest overall reduction in psychological distress.

**Findings:**

Sixty-eight participants were enrolled (17 per group), with no losses to follow-up. B-GAD7 did not differ across groups (p = 0.908). Compared with Control, Yoga+Gita reduced anxiety immediately (p = 0.009), and Gita reduced anxiety at 45 days (p = 0.047). A clinically meaningful (≥4-point) GAD7 reduction at 45 days occurred in 17.6% (Yoga), 47.1% (Gita), 41.2% (Yoga+Gita), and 11.8% (Control) of the participants. Exploratory analyses suggested larger benefits among women and among those who continued practice.

**Interpretation:**

Yoga and Gita-learning, particularly in combination, were associated with reductions in psychological distress among HCWs.

**Trial registration:**

Prospectively registered with the CTRI (CTRI/2021/10/037365).

## 1. Introduction

To effectively combat public health challenges, it is important for the frontline workers to remain healthy. According to the World Health Organization (WHO), the psychological wellbeing of healthcare workers (HCWs) is as important as their physical health [1]. Globally, HCWs reported increased mental health problems during the coronavirus disease (COVID-19) pandemic [2].

The WHO advised the HCWs to seek social support for maintaining mental wellbeing [1]. The managers of health facilities were instructed to facilitate access to mental health support services for staff [1]. Thus, the need for mental health support for HCWs has been recognized; however, no specific strategy has been devised by the medical fraternity to address the HCWs’ mental health concerns [3]. Patel et al. recommend engaging in meditation and yoga to manage stress in HCWs, and approaching therapists if required [4]. Li et al. discuss how online counselling is being adopted to provide mental health support to HCWs and patients in China [5]. Another study summarizes some of the practices adopted by different countries to address the psychological impact of COVID-19 on HCWs, including measures like peer support programs, online courses on common psychological problems, facilitating counselling at staff rest areas, and a dedicated helpline to provide advice and psychological support to the HCWs [6]. Ho et al. suggest shorter and rotating shifts and regular breaks for frontline HCWs [7]. Kar et al. recommend healthy lifestyle measures and pursuing hobbies [3].

However, despite psychological distress, most HCWs decline seeking help [2,8,9], rendering the mental health support services unproductive. Research suggests that HCWs do not access mental health services due to unrealistic expectations of themselves, denial and minimization of symptoms, and stigma [2,9,10]. Therefore, mere creation of mental health support systems may be of limited help, and facilitation of their access by the managers may be difficult as HCWs do not seek help.

Culturally oriented health services, including mental health services, are known to improve quality of care [10]. To make mental health services culturally appropriate, socio-constructive approaches that emphasize the cultural context of an individual’s experience and reality have been adopted [11]. Despite recognizing the need for culturally sensitive mental health services, there is no specific guidance from the international medical fraternity on culturally tailoring mental health services to HCWs. This is especially problematic for a country like India, where the cultural context of the patients and the HCWs differs considerably from the West. The bulk of the research on mental health has been carried out in Western countries, and it cannot be said with certainty how successfully the findings can applied to other cultures [12]. Therefore, evidence-based culturally-sensitive programs must be designed [12].

During the peak of the first wave of the COVID-19 pandemic in India, we instituted mental support measures, such as access to a confidential counselling and psychology service and instituting a peer support group, for HCWs at our hospital. However, the uptake of these services was low—out of 150 HCWs working at our hospital, only 3 HCWs accessed the counselling and psychology service and 5 HCWs participated in the peer support group between 16, September 2020 and 17, March 2021—and it was unviable for our hospital to continue providing these services. Subsequent research conducted at our hospital revealed that there was significant stigma attached to seeking mental health support and that most HCWs turned to spirituality as a coping mechanism [2]. Thus, recognizing the value of culturally-oriented services, which may be rooted in spirituality in order to enhance uptake, herein, we attempt to validate the postulations of a rather oriental and emic approach by Das & Behura based on imparting the teachings of the Bhagavad Gita—a Hindu scripture—to the HCWs alongside conventional measures [13]. The Bhagavad Gita is a popular source of knowledge and wisdom. The narration of the Gita, set in the middle of a battlefield, provides direction and lifts the spirits of a warrior (Arjuna), who is waging an inner battle alongside the external one. Das & Behura hypothesized that the Bhagavad Gita can help the HCWs, just like it helped Arjuna, despite the cause of distress of the HCWs having no moral bearings, unlike the one that plagued Arjuna [13]. The specific hypotheses evaluated in this study are as follows:

(1) Practicing yoga, learning the Bhagavad Gita, or combining the two will lead to significant reductions in psychological distress compared to a control group; (2) learning the Bhagavad Gita will have more lasting effects on stress reduction than Yoga alone; and (3) Yoga and Bhagavad Gita will act synergistically to provide both immediate and long-term stress reductions.

## 2. Methods

This randomized controlled trial is reported in accordance with the CONSORT 2010 checklist, which is provided in the Supporting Information (See S1 File).

### 2.1. Study setting

The study was conducted at Sri Jagannath Healthcare And Research Center (SJHRC), a secondary hospital in Dhanbad, Jharkhand, India, between the second and third waves of the COVID-19 pandemic in India. Jharkhand has a doctor–patient ratio of 1:8200. Participant recruitment commenced on 11^th^ October, 2021 and was completed on 23^rd^ October, 2021.

### 2.2. Ethical considerations, participant recruitment, and study design

The study and all the procedures were approved by the SJHRC – Independent Ethics Committee (approval number: SJHRC – NI/21/OCT/07). The trial was prospectively registered on the Clinical Trials Registry of India (CTRI/2021/10/037365). A call for participants was posted on the internal mail system and notice board of SJHRC, along with all relevant information on the study. The study remained open for enrollment for 2 weeks. When a HCW showed interest in participating in the study, they were invited to an interview with the research team where the research team again explained the objectives and methods of the study and the HCWs clarified any questions they had about the study; then the HCWs were assessed against the inclusion and exclusion criteria, and written informed consent was obtained from those deemed eligible for the study. The inclusion criteria were age > 18 years, current employment at SJHRC as an HCW, and availability for the duration of the interventions and follow-up. The exclusion criterion was the presence of any pre-existing diagnosed mental health condition; this was to ensure that the study measured the impact of the interventions on psychological distress arising from work-related stress during the pandemic and not underlying disorders.

Upon returning the signed consent form to the research office, the participants were randomized in a 1:1:1:1 ratio using a computer-generated random sequence. The sequence, containing participant identification numbers (PINs) and corresponding group assignments, was generated in advance by a researcher not involved in recruitment and placed into sequentially numbered, opaque, sealed envelopes that were handled only by research staff uninvolved in enrollment. The sealed envelopes were placed in a container prior to enrolment. After written informed consent was obtained, each participant selected one envelope from the container, which was then opened to reveal their group assignment. This procedure ensured both random sequence generation and allocation concealment at the time of enrollment. All data was collected anonymously using the PIN as the only identifier. The consent forms and a document linking the names of the participants to their PIN was kept under lock and key at the research office of SJHRC; the research team of this study did not have access to this document.

A prospective, randomized controlled repeated measures design was employed in this study. The trial protocol was registered on the OSF registry (https://doi.org/10.17605/OSF.IO/PXG93) and is provided in the Supporting Information (see S2 File). The novel intervention (imparting the teachings of the Bhagavad Gita alongside practicing yoga) was tested against a standard intervention (practicing yoga). We also tested the efficacy of imparting the teachings of the Bhagavad Gita as a standalone intervention. Thus, there were four study arms: Control—the participants received no intervention, Yoga—the participants attended an hour-long yoga session every day for one week, Gita—the participants attended an hour-long Bhagavad Gita session every day for one week, Yoga+Gita—the participants attended an hour-long session where they were made to practice yoga for the first 30 mins, and the Bhagavad Gita was taught over the remaining 30 mins.

Verses from the second chapter of the Bhagavad Gita were taught and their meaning was explained by trained teachers of International Society for Krishna Consciousness (ISKCON), Dhanbad, using the text Bhagavad-Gita As It Is as part of the intervention to participants in the Gita and Yoga+Gita groups. Chapter 2 was selected because it synthesizes core teachings on detachment, duty, and mind-control, providing a practical cognitive framework for stress regulation and resilience. Printed handouts of the verses were provided, the same instructor delivered all sessions, and participants were encouraged to review the verses during the 45-day follow-up period. Each 60-minute Yoga session (Yoga group) followed a fixed, standardized format consisting of approximately 30 minutes of asana postures, 15 minutes of pranayama, and 15 minutes of guided meditation. The 30-minute Yoga sessions in the Yoga+Gita arm followed the same sequence, with each component proportionally reduced in duration. Participants in the Yoga and Yoga+Gita groups were taught and made to practice the following poses and breathing exercises by trained teachers of Ayush and Skill Development Research Institute, Dhanbad: Bhadrasana (Gracious Pose), Mandukasana (Frog Pose), Shawasana (Corpse pose), Paschimottanasana (Seated Forward Bend Pose), Naadishodhana Pranayama (Alternate nostril breathing), Bhramari Pranayama (Bee breath), Sheetkari Pranayama (Hissing Breath), Sheetali Pranayama (Cooling Breath), and Dhyana (Meditation). The same instructors conducted all Yoga sessions throughout the intervention period.

### 2.3. Randomization and masking

Participants were randomized as described in Section 2.2, using a computer-generated allocation sequence with concealment via sequentially numbered, opaque, sealed envelopes. The research teams were separated into three groups: Team 1 handled recruitment and enrollment; Team 2 coordinated the interventions and collected outcome data; and Team 3 conducted data analysis. The three teams did not have contact with each other. Due to the behavioral nature of the interventions, participants and instructors were aware of group assignments. However, personnel conducting data analysis (Team 3) were blinded to treatment allocation.

### 2.4. Measures

The Generalised Anxiety Disorder-7 (GAD7) was used to measure psychological distress as it is sensitive to change over a short period [14]. It has also been used by previous studies to measure psychological outcomes in HCWs during the COVID-19 pandemic [15]. One week after the last participant had enrolled in the study, the GAD7 was administered to all participants; this was the baseline assessment (B-GAD7). Along with the B-GAD7, demographic data (age, gender, and education) was also collected from the participants. The pertinent interventions were started on the next day for the three intervention groups. On the eighth (1 day post intervention) and the fifty-second (45 days post-intervention) days after the start of the interventions, the GAD7 was again administered to all groups, including the Control group; these were called the immediate post-intervention GAD7 (IPI-GAD7) and the delayed post-intervention GAD7 (DPI-GAD7), respectively. While collecting the DPI-GAD7 data, the participants in the intervention groups were also asked a single yes/no question: if they had continued the practices taught during the intervention; no data were collected on frequency, duration, or fidelity of continued practice.

### 2.5. Statistical analysis

PASS 2020, v20·0·6 (NCSS LLC, UT, USA) was used to calculate the sample size based on a two-sided two-sample t-test of mean change in GAD7 scores for an α of 5%, 1-β of 80%, assuming a minimum clinically important difference of 4 points for the GAD7 [16], a standard deviation (SD) of 3·78 based on a previous study of one of the interventions of interest [17], and a dropout rate of 15%; this model was used for power only. This calculation targeted a two-group intervention-versus-control comparison and did not incorporate multiplicity adjustment for all pairwise comparisons among the four arms. Data were analyzed using Python 3·9. The Shapiro–Wilk test was used to test the normality of the data for age and for GAD7 within each group at each time point (baseline, IPI, DPI). Age and GAD7 scores were not normally distributed. Between groups at baseline, age was compared with Kruskal-Wallis, while gender and education, being categorical, were compared using χ² tests. To compare GAD7 scores within the groups over time and across the groups, the Friedman and Kruskal–Wallis tests were utilized, respectively. Following a significant Friedman test result, pairwise comparisons were conducted using the Conover-Friedman post-hoc test. For these Conover-Friedman post-hoc comparisons, p-values were adjusted using a Bonferroni correction (α_adj = 0.05/k, where k is the number of pairwise comparisons within each test family). If the Kruskal–Wallis test indicated significant differences, Dunn’s pairwise tests with Bonferroni-adjusted two-sided p-values were used for pairwise comparisons. To measure the effect size of the differences in GAD7 values between groups, η² was reported (for Kruskal-Wallis). The Kendall’s W was calculated for Friedman to measure the effect size of the differences in GAD7 values within groups over time. We summarized the proportion achieving the MCID (≥4-point reduction from Baseline to DPI). For exploratory estimation of clinical effect, odds ratios and 95% confidence intervals were calculated using exact methods for 2 × 2 contingency tables (conditional maximum likelihood estimation), given the small cell counts, comparing MCID responder status in each intervention arm versus control. Percent change was computed per participant as 100×(DPI–Baseline)/Baseline (participants with Baseline = 0 excluded), summarized as median [IQR]. Additionally, to determine the effect of continued practice of the intervention, which is pivotal for understanding the sustained impact of the interventions on psychological wellbeing, we divided each group into subgroups based on whether the participants continued with the practices taught during the intervention, and employed the Mann–Whitney U test (exploratory) to compare the DPI-GAD7 scores among the subgroups. Exploratory subgroup analyses were also conducted based on gender and educational attainment, and the Mann–Whitney U test was utilized to compare the B-GAD7, IPI-GAD7, and DPI-GAD7 scores between the subgroups. Given limited power, all continued-practice and subgroup analyses were exploratory and not adjusted for multiple comparisons; their results should therefore be interpreted as hypothesis-generating, whereas primary post-hoc comparisons (Dunn’s and Conover–Friedman tests) were Bonferroni-adjusted and are reported with corresponding effect sizes (η² and Kendall’s W). A p-value <0·05 was deemed significant for all statistical tests. Between-group tests used all available observations at each timepoint; Friedman/Conover–Friedman used complete cases with all three timepoints within arm; MCID analysis required both Baseline and DPI-GAD7 scores.

## 3. Results

### 3.1. Timeline of interventions

The B-GAD7 was administered on 1^st^ November, 2021 and the interventions ran between 2^nd^ November, 2021 and 8^th^ November, 2021. The IPI-GAD7 and DPI-GAD7 were administered on 9^th^ November, 2021 and 23^rd^ December, 2021, respectively. There were 17,948 COVID-19-related deaths in India between 1^st^ November, 2021 and 23^rd^ December, 2021 [18].

### 3.2. Study participants

**Fig 1:**
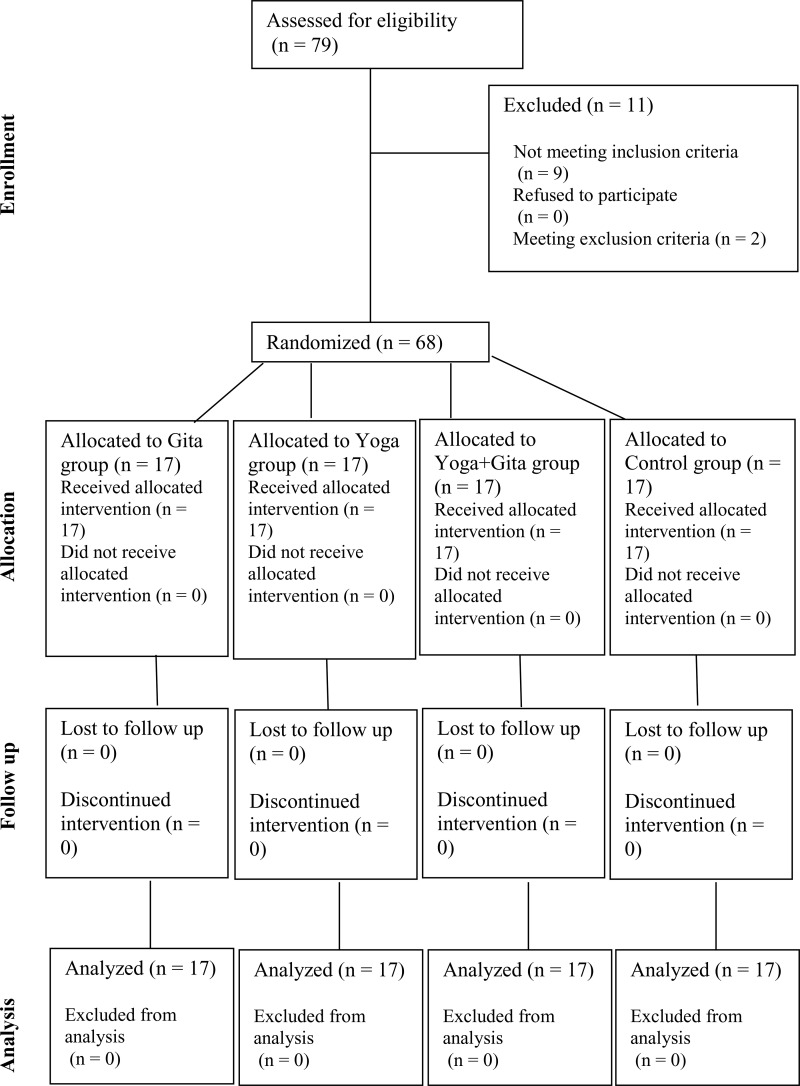
CONSORT flow diagram showing the number of participants assessed for eligibility (n = 79), excluded (n = 11), randomized (n = 68), allocated to each arm, followed up, and analyzed (n = 17 per group).

The sample size calculation revealed that 68 participants, with 17 participants per group, were required to yield an α of 5% and 1-β of 80%. There were no losses or exclusions after randomization. All participants were included in the analysis and the analysis was by original assigned groups. All participants had at least a Bachelor’s degree. There were no statistically significant differences between the groups at baseline in age (Kruskal–Wallis p = 0·938), educational attainment (χ² p = 0·891), or gender (χ² p = 0·706). The demographic characteristics of the participants is presented in Table 1. The CONSORT flowchart of the study is shown in Fig 1.

**Table 1:** Participant demographic characteristics.

Group	N	Age (Median [IQR]); years	Gender, n (%)		Education, n (%)	
			Men	Women	Bachelors	Masters or higher
**Yoga**	17	29·000 [24·000, 49·000]	11 (64·700%)	6 (35·300%)	9 (52·900%)	8 (47·100%)
**Gita**	17	30·000 [26·000, 52·000]	11 (64·700%)	6 (35·300%)	10 (58·800%)	7 (41·200%)
**Yoga+Gita**	17	37·000 [30·000, 45·000]	8 (47·100%)	9 (52·900%)	10 (58·800%)	7 (41·200%)
**Control**	17	36·000 [30·000, 46·000]	10 (58·800%)	7 (41·200%)	8 (47·100%)	9 (52·900%)

*IQR, interquartile range.*

### 3.3. Changes in GAD7 values

Table 2 shows the median (interquartile range) of the GAD7 scores over time in each group. The results illustrate a marked reduction in anxiety levels for participants in the Gita and Yoga+Gita groups.

**Table 2:** GAD7 values by group over time.

Group	B-GAD7	IPI-GAD7	DPI-GAD7	%Change (Baseline to 45 Days) (Median %Change [IQR])	MCID Responders	
	Median [IQR]	Median [IQR]	Median [IQR]		n	%
**Yoga**	6·000 [4·000, 9·000]	5·000 [3·000, 7·000]	5·000 [2·000, 6·000]	−30·000% [-50·000%, 0·000%]	3	17·6%
**Gita**	7·000 [5·000, 9·000]	4·000 [3·000, 6·000]	3·000 [2·000, 4·000]	−50·000% [-70·000%, -33·333%]	8	47·1%
**Yoga+Gita**	6·000 [4·000, 7·000]	3·000 [2·000, 5·000]	4·000 [1·000, 5·000]	−50·000% [-66·667%, -20·000%]	7	41·2%
**Control**	7·000 [4·000, 9·000]	6·000 [4·000, 8·000]	7·000 [4·000, 9·000]	−8·333% [-33·333%, 25·000%]	2	11·8%

*MCID, minimum clinically important difference (Responders are those with MCID >=4); GAD7, generalised anxiety disorder-7; B-GAD7, baseline GAD7; IPI-GAD7, immediate post-intervention GAD7; DPI-GAD7, delayed post-intervention GAD7; IQR, interquartile range.*

*MCID Responders are participants with ≥4-point reduction in GAD7 from Baseline to DPI (Day 52), denominator for calculating % = participants with both Baseline and DPI measurements. %Change is computed per participant as 100×(DPI−Baseline)/Baseline (participants with Baseline = 0 excluded), then summarized as median [IQR].*

#### 3.3.1. Across groups comparison.

**Fig 2:**
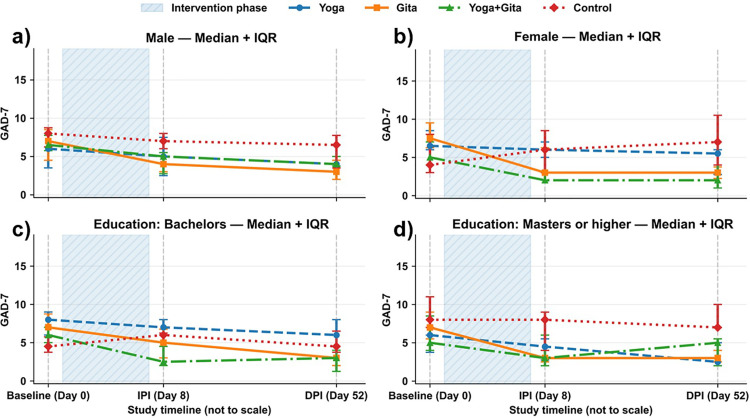
Median (IQR) GAD7 scores over time by study group, stratified by gender and education level (exploratory). Scores are shown at baseline (Day 0), immediately post-intervention (Day 8), and 45 days post-intervention (Day 52). The shaded region indicates the intervention period.

The Kruskal–Wallis test showed no significant differences in the B-GAD7 values across the groups (p = 0·908). However, it showed significant differences in the IPI-GAD7 (p = 0·011; η^2^ = 0·166; Dunn’s test significant between the Yoga+Gita and Control groups, p = 0·009) and DPI-GAD7 (p = 0·025; η^2^ = 0·140; Dunn’s test significant between the Gita and Control groups, p = 0·047). Following Cohen’s convention, η² values ≥0·14 were interpreted as large effects, indicating a meaningful between-group separation at both post-intervention timepoints. The detailed results of the Dunn’s test are shown in Table 3. Compared with Control, the odds of achieving MCID were higher in the Gita group (OR=6.67, 95% CI 1.15–38.60) and in the Yoga+Gita group (OR=5.25, 95% CI 0.90–30.62), whereas the Yoga group showed little difference (OR=1.61, 95% CI 0.23–11.09). Confidence intervals were wide due to the small sample size. The group medians over time is presented in Fig 2.

**Table 3:** Results of the Dunn’s test.

Timepoint	Comparison	Dunn’s test p-value	Significant Difference
IPI-GAD7	Yoga vs Gita	1·000	No
IPI-GAD7	Yoga vs Yoga+Gita	0·317	No
IPI-GAD7	Yoga vs Control	1·000	No
IPI-GAD7	Gita vs Yoga+Gita	1·000	No
IPI-GAD7	Gita vs Control	0·153	No
IPI-GAD7	Yoga+Gita vs Control	0·009	Yes
DPI-GAD7	Yoga vs Gita	1·000	No
DPI-GAD7	Yoga vs Yoga+Gita	1·000	No
DPI-GAD7	Yoga vs Control	0·944	No
DPI-GAD7	Gita vs Yoga+Gita	1·000	No
DPI-GAD7	Gita vs Control	0·047	Yes
DPI-GAD7	Yoga+Gita vs Control	0·057	No

*GAD7, generalised anxiety disorder-7; B-GAD7, baseline GAD7; IPI-GAD7, immediate post-intervention GAD7; DPI-GAD7, delayed post-intervention GAD7*

#### 3.3.2. Within group comparison.

The Friedman test showed no significant differences in the GAD7 values over time in the Control group (p = 0·779). However, it showed significant differences in the Yoga (p = 0·001; Kendall’s W = 0·422), Gita (p < 0·001; Kendall’s W = 0·402), and Yoga+Gita (p < 0·001; Kendall’s W = 0·410) groups. As per Cohen’s convention, the Kendall’s W values around 0·40 were interpreted as reflecting a moderate-to-large degree of concordance in GAD-7 change over time within these intervention arms. The Conover test showed significant differences between B-GAD7 and DPI-GAD7 in the Yoga (p = 0·007) and Gita (p = 0·003) groups, and between B-GAD7 and IPI-GAD7 (p = 0·012) and B-GAD7 and DPI-GAD7 (p = 0·011) in the Yoga+Gita group. The detailed results of the Conover test are shown in Table 4.

**Table 4:** Results of the Conover test.

Group	Comparison	Conover’s Post-hoc p-values	Significant Difference
Yoga	B-GAD7 vs. IPI-GAD7	0·001	Yes
Yoga	B-GAD7 vs. DPI-GAD7	0·001	Yes
Yoga	IPI-GAD7 vs. DPI-GAD7	1·000	No
Gita	B-GAD7 vs. IPI-GAD7	0·000	Yes
Gita	B-GAD7 vs. DPI-GAD7	0·000	Yes
Gita	IPI-GAD7 vs. DPI-GAD7	0·355	No
Yoga+Gita	B-GAD7 vs. IPI-GAD7	0·000	Yes
Yoga+Gita	B-GAD7 vs. DPI-GAD7	0·000	Yes
Yoga+Gita	IPI-GAD7 vs. DPI-GAD7	1·000	No

*GAD7, generalised anxiety disorder-7; B-GAD7, baseline GAD7; IPI-GAD7, immediate post-intervention GAD7; DPI-GAD7, delayed post-intervention GAD7*

### 3.4. Subgroup analysis

All subgroup analyses presented below were exploratory and were not powered for confirmatory inference.

#### 3.4.1. By gender.

The data revealed that women experienced a more pronounced decrease in GAD-7 scores, especially within the Gita and Yoga+Gita groups. The median B-GAD-7, IPI-GAD7, and DPI-GAD7 scores along with the IQRs for both men and women across all groups are shown in Table 5.

**Table 5:** GAD7 values by gender and group over time.

Group	Gender	B-GAD7 (Median [IQR])	Mann–Whitney U p-value	IPI-GAD7 (Median [IQR])	Mann–Whitney U p-value	DPI-GAD7 (Median [IQR])	Mann–Whitney U p-value	%Change (Baseline to 45 Days) (Median %Change [IQR])	MCID Responders	
									n	%
Yoga	Men	6·000 [3·500, 8·500]	0·838	5·000 [2·500, 7·500]	0·959	4·000 [2·000, 6·500]	0·839	−30·000% [-50·000%, -8·333%]	1	9·1%
Yoga	Women	6·500 [6·000, 8·500]	··	6·000 [5·000, 7·000]	··	5·500 [2·750, 6·000]	··	−29·365% [-48·611%, -3·571%]	2	33·3%
Gita	Men	7·000 [4·500, 8·500]	0·544	4·000 [3·000, 5·500]	0·542	3·000 [2·000, 4·500]	0·642	−50·000% [-57·143%, -31·667%]	4	36·4%
Gita	Women	7·500 [6·250, 9·500]	··	3·000 [2·000, 6·250]	··	3·000 [2·250, 3·750]	··	−70·714% [-76·786%, -55·000%]	4	66·7%
Yoga+Gita	Men	6·500 [5·750, 7·750]	0·383	5·000 [2·750, 5·500]	0·067	4·000 [3·750, 5·000]	0·242	−41·667% [-57·857%, -19·167%]	3	37·5%
Yoga+Gita	Women	5·000 [4·000, 7·000]	··	2·000 [2·000, 3·000]	··	2·000 [1·000, 4·000]	··	−50·000% [-85·714%, -40·000%]	4	44·4%
Control	Men	8·000 [6·250, 8·750]	0·169	7·000 [6·000, 8·000]	0·489	6·500 [3·500, 7·750]	0·493	−14·583% [-38·333%, 0·000%]	2	20·0%
Control	Women	4·000 [3·000, 8·000]	··	6·000 [3·000, 8·500]	··	7·000 [4·000, 10·500]	··	27·273% [-14·167%, 83·333%]	0	0·0%

*MCID, minimum clinically important difference (Responders are those with MCID >=4); GAD7, generalised anxiety disorder-7; B-GAD7, baseline GAD7; IPI-GAD7, immediate post-intervention GAD7; DPI-GAD7, delayed post-intervention GAD7; IQR, interquartile range.*

*MCID Responders are participants with ≥4-point reduction in GAD7 from Baseline to DPI (Day 52), denominator for calculating % = participants with both Baseline and DPI measurements. %Change is computed per participant as 100×(DPI−Baseline)/Baseline (participants with Baseline = 0 excluded), then summarized as median [IQR].*

Men in all three groups showed improvements in GAD7 scores over time (Friedman test: Yoga, p = 0·009; Gita, p = 0·003; Yoga+Gita, p = 0·002). However, the control group remained largely unchanged. Women demonstrated a more substantial reduction in GAD7 scores, particularly in the Gita (Friedman test, p = 0·006) and Yoga+Gita (Friedman test, p = 0·002) groups. However, the Yoga group did not show a significant difference (Friedman test, p = 0·108). Notably, the control group showed an increase in DPI-GAD7 scores in women, although not significant (Friedman test, p = 0·618), contrasting with the intervention groups’ outcomes.

#### 3.4.2. By Education Level.

The median B-GAD-7, IPI-GAD7, and DPI-GAD7 scores for participants with a Bachelors degree and those with a Masters degree or higher, along with the IQRs, across all groups are shown in Table 6.

**Table 6:** GAD7 values by educational level and group over time.

Group	Education Level	B-GAD7 (Median [IQR])	Mann–Whitney U p-value	IPI-GAD7 (Median [IQR])	Mann–Whitney U p-value	DPI-GAD7 (Median [IQR])	Mann–Whitney U p-value	%Change (Baseline to 45 Days) (Median %Change [IQR])	MCID Responders	
									n	%
**Yoga**	Bachelors	8·000 [6·000, 9·000]	0·129	7·000 [5·000, 8·000]	0·224	6·000 [4·000, 8·000]	0·132	−20·000% [-50·000%, 0·000%]	1	11·1%
**Yoga**	Masters or higher	6·000 [3·750, 6·000]	··	4·500 [2·750, 5·500]	··	2·500 [2·000, 5·000]	··	−38·889% [-50·000%, -12·500%]	2	25·0%
**Gita**	Bachelors	7·000 [4·500, 8·750]	0·844	5·000 [3·000, 6·750]	0·430	3·000 [2·000, 3·750]	0·725	−50·000% [-66·786%, -37·500%]	4	40·0%
**Gita**	Masters or higher	7·000 [5·500, 9·000]	··	3·000 [2·500, 4·000]	··	3·000 [2·500, 4·000]	··	−57·143% [-69·286%, -41·667%]	4	57·1%
**Yoga+Gita**	Bachelors	6·000 [5·000, 7·000]	0·883	2·500 [2·000, 4·500]	0·393	3·000 [1·250, 4·000]	0·277	−53·571% [-65·000%, -35·000%]	4	40·0%
**Yoga+Gita**	Masters or higher	5·000 [4·000, 8·500]	··	3·000 [2·000, 6·000]	··	5·000 [2·000, 5·500]	··	−46·667% [-75·000%, -17·143%]	3	42·9%
**Control**	Bachelors	4·500 [3·750, 5·750]	0·037	6·000 [5·000, 6·250]	0·263	4·500 [3·750, 6·500]	0·192	−10·000% [-35·000%, 27·083%]	0	0·0%
**Control**	Masters or higher	8·000 [7·000, 11·000]	··	8·000 [4·000, 9·000]	··	7·000 [7·000, 10·000]	··	−8·333% [-16·667%, 16·667%]	2	22·2%

*MCID, minimum clinically important difference (Responders are those with MCID >=4); GAD7, generalised anxiety disorder-7; B-GAD7, baseline GAD7; IPI-GAD7, immediate post-intervention GAD7; DPI-GAD7, delayed post-intervention GAD7; IQR, interquartile range.*

*MCID Responders are participants with ≥4-point reduction in GAD7 from Baseline to DPI (Day 52), denominator for calculating % = participants with both Baseline and DPI measurements. %Change is computed per participant as 100×(DPI−Baseline)/Baseline (participants with Baseline = 0 excluded), then summarized as median [IQR].*

For participants with a Bachelors degree (Yoga, p = 0·041; Gita, p < 0·001; Yoga+Gita, p < 0·001), as well as for those with a Masters degree or higher (Yoga, p = 0·023; Gita, p = 0·049; Yoga+Gita, p = 0·018), the Friedman test showed significant improvements in GAD7 scores over time in all three groups. However, the control group remained largely unchanged in the participants with a Bachelors degree as well as those with a Masters degree or higher (p = 0·531 and p = 0·402, respectively).

#### 3.4.3. By continued practice.

In general, participants who continued practicing the interventions tended to show lower DPI-GAD7 scores than those who did not continue practicing the interventions (Table 7); however, statistical significance (p = 0·033) was observed only in the Yoga+Gita group.

**Table 7:** Impact of continued practice on DPI-GAD7 scores.

Group	Practice Continued	N	DPI-GAD7 (Median [IQR])	Mann–Whitney U p-value	%Change (Baseline to 45 Days) (Median %Change [IQR])	MCID Responders	
						n	%
Yoga	Yes	12	4·500 [2·000, 6·250]	0·873	−37·222% [-51·389%, -16·071%]	3	25·0%
Yoga	No	5	6·000 [2·000, 6·000]	··	0·000% [-33·333%, 0·000%]	0	0·0%
Gita	Yes	6	3·000 [3·000, 3·750]	0·502	−53·571% [-66·786%, -50·000%]	4	66·7%
Gita	No	11	3·000 [2·000, 4·000]	··	−50·000% [-65·714%, -33·333%]	4	36·4%
Yoga+Gita	Yes	11	3·000 [1·000, 4·000]	0·033*	−60·000% [-87·302%, -45·000%]	6	54·5%
Yoga+Gita	No	6	5·000 [4·250, 5·750]	··	−18·333% [-40·000%, -14·881%]	1	16·7%

*MCID, minimum clinically important difference (Responders are those with MCID >=4); GAD7, generalised anxiety disorder-7; DPI-GAD7, delayed post-intervention GAD7; IQR, interquartile range.*

*MCID Responders are participants with ≥4-point reduction in GAD7 from Baseline to DPI (Day 52), denominator for calculating % = participants with both Baseline and DPI measurements. %Change is computed per participant as 100×(DPI−Baseline)/Baseline (participants with Baseline = 0 excluded), then summarized as median [IQR].*

## 4. Discussion

COVID-19 has posed serious challenges to the mental health of the HCWs [1,2,8]. Several interventions have been suggested to support the mental health of HCWs during the COVID-19 pandemic. Chiappetta et al. concluded that mindfulness-based interventions improved the mental wellbeing of the HCWs [19]. Another study showed that HCWs who had social support had better sleep quality, and prescribed employing psychotherapy teams to support the HCWs [20]. Yoga, relaxation, and meditation have also been recommended [4,15]. Some hospitals provided psychological support to HCWs through ensuring regular counsellor visits, training HCWs on how to relax, and encouraging leisure activities [21]. Hotlines for psychological assistance have been established to combat the mental health challenges of HCWs [22].

However, in a study of 300 HCWs with anxiety and depression, 59·8% participants said they would not consider seeking psychological support [8]. HCWs often feel ashamed of seeking help [23] and fear it may harm their reputation [9]. In India, mental health support systems for HCWs are largely lacking [24]. In our hospital, psychological support services were underutilized despite significant stress during the pandemic. Evidently, HCWs have a negative attitude towards mental health issues and are reluctant to seek help. Therefore, an intervention that does not appear like a psychological intervention at the outset can act as a workaround solution. Given prior findings that HCWs turned to spirituality as a coping mechanism during the COVID-19 pandemic [2], we sought to provide a spirituality-based intervention to support their mental health.

In this study, there were no significant differences in age, educational attainment, gender, and B-GAD7 scores, among the groups at baseline, suggesting that any observed effects of the interventions are less likely to be confounded by these baseline characteristics. The results showed significant reductions in GAD7 scores from baseline to immediately post-intervention and from baseline to 45 days post-intervention in all intervention groups, underscoring the effectiveness of Yoga and the Bhagavad Gita teachings, alone and in combination, in mitigating stress. Contrarily, the lack of changes in GAD7 scores over time in the control group reinforce that the improvements in the GAD7 scores in the intervention groups are directly attributable to the interventions. Moreover, the sustained reductions in GAD7 scores 45 days post-intervention, particularly in the Gita and Yoga+Gita groups, underscore the lasting impact of these interventions on reducing stress. The large effect sizes for IPI-GAD7 and DPI-GAD7 indicate that the Yoga+Gita and Gita-alone interventions have a substantial practically significant impact on reducing stress among HCWs compared to the control group, highlighting the potential for integrating these traditional approaches into therapeutic contexts.

Interestingly, only the Yoga+Gita group showed a significant reduction in IPI-GAD7 scores and only the Gita group showed a significant reduction in DPI-GAD7 scores when the GAD7 scores at each timepoint were compared across the groups. This result underscores the complementary nature of Yoga and Gita. While Yoga offers immediate physical relief through breath control and relaxation, this alone may not be sufficient to produce significant stress reduction in a short span of 1 week. Indeed, most Yoga-alone interventions for stress reduction span a longer period of 4 weeks [25]. The Bhagavad Gita provides deeper emotional and cognitive benefits that foster long-term resilience. The combination of Yoga and Gita leads to both immediate and sustained stress reduction, as Yoga addresses body discomfort and tension by shifting the balance from the sympathetic to parasympathetic nervous system [26], and the Gita’s teachings promote a shift in mindset and emotional stability. The Gita alone works more gradually, explaining its impact after 45 days, as spiritual and cognitive frameworks take time to reshape attitudes and reduce distress. Hence, there is a need to integrate physical and spiritual practices for comprehensive mental health benefits. The Kendall’s W values indicate that the interventions lead to moderate–strong reductions in psychological distress over time within each intervention group, validating their effectiveness.

In exploratory responder analyses, the odds of achieving a clinically meaningful reduction in GAD7 (≥4 points) were higher in the Gita arm compared with Control (OR = 6.67, 95% CI 1.15–38.60), with a similarly elevated but imprecise estimate observed in the Yoga+Gita arm (OR = 5.25, 95% CI 0.90–30.62). In contrast, Yoga alone showed little difference relative to Control (OR = 1.61, 95% CI 0.23–11.09). These effect size estimates suggest that inclusion of structured Bhagavad Gita-based cognitive reframing may increase the likelihood of clinically meaningful anxiety reduction among healthcare workers. However, confidence intervals were wide, reflecting limited precision due to the small sample size. The study was not powered for categorical responder outcomes; therefore, these findings should be interpreted as exploratory and hypothesis-generating rather than definitive evidence of superiority. Larger trials powered specifically for categorical responder outcomes are required to confirm these estimates.

Women demonstrated a more substantial reduction in GAD7 scores, particularly in the Gita and Yoga+Gita groups, suggesting that while both men and women benefit from these interventions, women might experience more significant improvements. This result is similar to that of a previous study which revealed that women showed a greater reduction in negative affect than men in response to meditation [27]. Therefore, considering gender as a factor in the design and evaluation of religious/spiritual interventions (RSIs) may be important. In terms of educational attainment, there were no differences in the responses of any group to the interventions. Thus, educational attainment may not influence the effectiveness of Yoga and Gita, alone and in combination, in mitigating stress. In general, participants who continued practicing the interventions tended to show lower DPI-GAD7 scores than those who did not; however, significance was reached only in the Yoga+Gita group, suggesting a potentially synergistic effect of continuing both practices.

RSIs can improve mental wellbeing by giving people a sense of purpose and acting as a coping mechanism to mitigate stress. Hook et al. [28] have opined that for psychological problems, RSIs may be more effective than secular therapies and even some drug treatments. Several studies on RSIs for mental health conditions have demonstrated a benefit of RSIs, especially at 1–3 months of follow-up [29,30]. Similar to these studies, our findings also show a significant benefit of Yoga and Gita, alone and in combination, on the mental wellbeing of HCWs, both immediately after the intervention and after 45 days. However, these previous studies on RSIs involved only Christian, Islamic, or Jewish practices and participants. Our study, on the contrary, examines Hindu RSIs (practicing Yoga and learning the Bhagavad Gita) and all our participants, except one, were practicing Hindus. Another main distinction of our study is that our RSI was relatively short, lasting 1 week, but showed significant sustained beneficial effects. Furthermore, to the best of our knowledge, this is the first study to explore the effectiveness of RSIs to support the mental health of HCWs.

The limitations of the study include a relatively smaller sample size due to challenges in recruiting HCWs at a time when they were already working in overstretched shifts. Because the sample size was calculated in PASS using the default two-group comparison, the trial may be underpowered to detect all pairwise differences across the three intervention arms and the control arm after multiplicity correction. Accordingly, some pairwise comparisons—particularly among intervention arms—may be subject to type II error. Subgroup and continued-practice analyses were exploratory and unadjusted for multiple comparisons, and therefore carry an increased risk of type I error. Consequently, the findings have limited external generalizability beyond similar single-center, secondary-care hospital settings and comparable HCW populations. Although linear mixed models or generalized estimating equations would model longitudinal correlation more efficiently, given the small sample size and the multi-arm repeated-measures design, mixed-model or generalized estimating equation estimates—particularly for group-by-time effects—would be imprecise and potentially unstable; we therefore used nonparametric tests given the observed non-normality. To address this limitation, our ongoing larger trial of these interventions in a different target population is powered specifically for mixed-model analysis [31].

Because of the nature of the interventions, participants were aware of their group assignment, which may have introduced expectancy or performance bias, particularly in the spiritually based intervention arms. However, outcome assessors and data analysts were blinded to group allocation, and participants were informed that all interventions were expected to reduce stress, which may have mitigated differential expectancy effects across groups. Adherence (‘continued practice’) was measured only with a single self-reported yes/no question and did not capture how often, how long, or how accurately participants practiced; this coarse measure may misclassify adherence and limits causal interpretation of sustained effects. Future studies should incorporate objective or structured adherence tracking (e.g., practice logs, digital monitoring, or instructor verification). In addition, use of a usual-care control does not account for nonspecific attention effects; future studies may benefit from including an attention-matched comparator arm.

Additionally, the sample is not broadly representative as it largely consists of participants belonging to the Hindu Religion, thereby limiting the applicability of the evaluated interventions. Analogous spiritual texts or secular contemplative practices in non-Hindu contexts may offer similar cognitive-emotional benefits and warrant systematic evaluation in future trials. Furthermore, the study may be affected by recruitment bias as HCWs who chose to participate in the study may be inherently spiritual/religious. Moreover, the inherent religiosity/spirituality of the participants may have confounded the results, and because we could not measure the baseline inherent religiosity/spirituality of the participants, we could not account for this potential confounder in our analyses. It remains to be seen how these interventions affect the HCWs belonging to other faiths and those who are agnostic to religion and spirituality, before generalizing these findings to all HCWs. We aim to flesh out the impact of the baseline spiritual/religious beliefs of the participants on the effectiveness of RSIs in a future study.

## 5. Conclusion

Psychological support systems have gone largely underutilized by HCWs, necessitating novel approaches to supporting their mental wellbeing. The findings herein underscore the significant positive impact of learning the Bhagavad Gita, alone and in combination with practicing Yoga, on the mental wellbeing of HCWs, suggesting that these approaches may offer effective strategies for managing psychological distress.

## Supporting information

S1 File CONSORT 2010 checklist of information to include when reporting a randomised trial. (DOCX)

S2 File Study protocol v4 (13.09.2021). (DOCX)

## References

[1] World Health Organization. Mental health and psychosocial considerations during the COVID-19 outbreak. 2020. https://www.who.int/docs/default-source/coronaviruse/mental-health-considerations.pdf?sfvrsn=6d3578af_2

[2] Konduru L, Das N, Kothari-Speakman G, Behura AK. Experiencing the COVID-19 pandemic as a healthcare provider in rural Dhanbad, India: An interpretative phenomenological analysis. PLoS One. 2022;17(8):e0273573. doi: 10.1371/journal.pone.0273573 36006877 PMC9409569

[3] Kar SK, Yasir Arafat SM, Kabir R, Sharma P, Saxena SK. Coping with Mental Health Challenges During COVID-19. Medical Virology: From Pathogenesis to Disease Control. Springer Singapore. 2020. p. 199–213. doi: 10.1007/978-981-15-4814-7_16

[4] Patel RS, Bachu R, Adikey A, Malik M, Shah M. Factors Related to Physician Burnout and Its Consequences: A Review. Behav Sci (Basel). 2018;8(11):98. doi: 10.3390/bs8110098 30366419 PMC6262585

[5] Li W, Yang Y, Liu Z-H, Zhao Y-J, Zhang Q, Zhang L, et al. Progression of Mental Health Services during the COVID-19 Outbreak in China. Int J Biol Sci. 2020;16(10):1732–8. doi: 10.7150/ijbs.45120 32226291 PMC7098037

[6] Selikowitz A, Rege S. Mental health challenges for healthcare workers during the COVID-19 pandemic – impact and management strategies. https://psychscenehub.com/psychinsights/mental-health-challenges-healthcare-workers-during-covid-19-pandemic-management-strategies/. 2020. Accessed 2024 October 23.

[7] Ho CS, Chee CY, Ho RC. Mental health strategies to combat the psychological impact of COVID-19 beyond paranoia and panic. Annals, Academy of Medicine, Singapore. 2020;49:1. 32200399

[8] Weibelzahl S, Reiter J, Duden G. Depression and anxiety in healthcare professionals during the COVID-19 pandemic. Epidemiol Infect. 2021;149:e46. doi: 10.1017/S0950268821000303 33557984 PMC7900665

[9] Galbraith ND, Brown KE, Clifton E. A survey of student nurses’ attitudes toward help seeking for stress. Nurs Forum. 2014;49(3):171–81. doi: 10.1111/nuf.12066 24392669

[10] Mutha S, Allen C, Welch M. Toward culturally competent care: A toolbox for teaching communication strategies. San Francisco, CA: Center for the Health Professions, University of California, San Francisco. 2002.

[11] Wallen J. Providing culturally appropriate mental health services for minorities. J Ment Health Adm. 1992;19(3):288–95. doi: 10.1007/BF02518993 10123308

[12] World Health Organization. Prevention and Promotion in Mental Health. 2001. https://www.who.int/mental_health/media/en/545.pdf

[13] Das N, Behura AK. Relevance of Bhagavad Gita for healthcare workers amidst the COVID -19 crisis. Asian J Psychiatr. 2021;56:102534. doi: 10.1016/j.ajp.2020.102534 33421693 PMC9760337

[14] Kertz S, Bigda-Peyton J, Bjorgvinsson T. Validity of the Generalized Anxiety Disorder-7 scale in an acute psychiatric sample. Clin Psychol Psychother. 2013;20(5):456–64. doi: 10.1002/cpp.1802 22593009

[15] Vizheh M, Qorbani M, Arzaghi SM, Muhidin S, Javanmard Z, Esmaeili M. The mental health of healthcare workers in the COVID-19 pandemic: A systematic review. J Diabetes Metab Disord. 2020;19(2):1967–78. doi: 10.1007/s40200-020-00643-9 33134211 PMC7586202

[16] Toussaint A, Hüsing P, Gumz A, Wingenfeld K, Härter M, Schramm E, et al. Sensitivity to change and minimal clinically important difference of the 7-item Generalized Anxiety Disorder Questionnaire (GAD-7). J Affect Disord. 2020;265:395–401. doi: 10.1016/j.jad.2020.01.032 32090765

[17] Sharma N, Sahni PS, Sharma US, Kumar J, Garg R. Effect of Yoga on the Stress, Anxiety, and Depression of COVID-19-Positive Patients: A Quasi-Randomized Controlled Study. Int J Yoga Therap. 2022;32(2022):Article 8. doi: 10.17761/2022-D-22-00013 35850136

[18] Worldometer. Countries where COVID-19 has spread. https://www.worldometers.info/coronavirus/countries-where-coronavirus-has-spread/. 2020. Accessed 2021 November 27.

[19] Chiappetta M, D’Egidio V, Sestili C, Cocchiara RA, Torre GL. Stress management interventions among healthcare workers using mindfulness: a systematic review. Senses and Sciences. 2018;5:517–49. doi: 10.14616/sands-2018-5-517549

[20] Xiao H, Zhang Y, Kong D, Li S, Yang N. The Effects of Social Support on Sleep Quality of Medical Staff Treating Patients with Coronavirus Disease 2019 (COVID-19) in January and February 2020 in China. Med Sci Monit. 2020;26:e923549. doi: 10.12659/MSM.923549 32132521 PMC7075079

[21] Chen Q, Liang M, Li Y, Guo J, Fei D, Wang L, et al. Mental health care for medical staff in China during the COVID-19 outbreak. Lancet Psychiatry. 2020;7(4):e15–6. doi: 10.1016/S2215-0366(20)30078-X 32085839 PMC7129426

[22] Kang L, Li Y, Hu S, Chen M, Yang C, Yang BX, et al. The mental health of medical workers in Wuhan, China dealing with the 2019 novel coronavirus. Lancet Psychiatry. 2020;7(3):e14. doi: 10.1016/S2215-0366(20)30047-X 32035030 PMC7129673

[23] Brooks SK, Gerada C, Chalder T. The specific needs of doctors with mental health problems: qualitative analysis of doctor-patients’ experiences with the Practitioner Health Programme. J Ment Health. 2017;26(2):161–6. doi: 10.1080/09638237.2016.1244712 27841030

[24] George S, Gupta N. Occupational mental health services for medical professionals: Relevance in the Indian context. Indian J Soc Psychiatry. 2020;36(2):98. doi: 10.4103/ijsp.ijsp_123_20

[25] Pilkington K, Kirkwood G, Rampes H, Richardson J. Yoga for depression: the research evidence. J Affect Disord. 2005;89(1–3):13–24. doi: 10.1016/j.jad.2005.08.013 16185770

[26] McCall T. Yoga as Medicine. New York, NY: Bantam *Dell*, *a division of Random House Inc*; 2007.

[27] Rojiani R, Santoyo JF, Rahrig H, Roth HD, Britton WB. Women Benefit More Than Men in Response to College-based Meditation Training. Front Psychol. 2017;8:551. doi: 10.3389/fpsyg.2017.00551 28473783 PMC5397480

[28] Hook JN, Worthington EL Jr, Davis DE, Jennings DJ 2nd, Gartner AL, Hook JP. Empirically supported religious and spiritual therapies. J Clin Psychol. 2010;66(1):46–72. doi: 10.1002/jclp.20626 19904806

[29] Miller WR, Forcehimes A, O’Leary MJ, LaNoue MD. Spiritual direction in addiction treatment: two clinical trials. J Subst Abuse Treat. 2008;35(4):434–42. doi: 10.1016/j.jsat.2008.02.004 18657945 PMC2600849

[30] Gonçalves JPB, Lucchetti G, Menezes PR, Vallada H. Religious and spiritual interventions in mental health care: a systematic review and meta-analysis of randomized controlled clinical trials. Psychol Med. 2015;45(14):2937–49. doi: 10.1017/S0033291715001166 26200715 PMC4595860

[31] Konduru L, Dahia SS, Kothari-Speakman G. Registered report: The effectiveness of a Bhagavad Gita intervention to reduce psychological distress in homeless people-A randomised controlled trial. PLoS One. 2024;19(11):e0310795. doi: 10.1371/journal.pone.0310795 39499690 PMC11537408

