# Supplementary material for: The stress reduction potential of Bhagavad Gita and Yoga for healthcare workers during the COVID-19 pandemic: A randomized controlled trial
Source: PLoS One. 2026 Apr 24;21(4):e0347320. doi: 10.1371/journal.pone.0347320 (PMC13108764; doi:10.1371/journal.pone.0347320)
Supplement: S2 File — (DOCX) [file pone.0347320.s002.docx]

**An Empirical Investigation of The Stress Reduction Potential of Bhagavad Gita and Yoga for Healthcare Workers During The COVID-19 Pandemic**

**S2: Study Protocol v4 (13.09.2021)**

Study questions

*1. If practicing yoga, learning the Bhagavad Gita, or combining both reduces psychological distress, then participants in these intervention groups will show significant reductions in GAD7 scores over time compared to their baseline scores.*

*2. If the interventions are more effective than no intervention, then the GAD7 scores will be significantly lower in the intervention groups compared to the control group at post-intervention assessments.*

*3. If learning the Bhagavad Gita results in more lasting stress reduction than practicing yoga alone, then the Gita group will have lower GAD7 scores at the delayed post-intervention assessment than the Yoga group.*

*4. If combining yoga and the Bhagavad Gita has a synergistic effect, then the Yoga+Gita group will experience greater immediate and sustained reductions in GAD7 scores than either intervention alone.*

*5. If no significant changes occur in the control group over time, then the GAD7 scores in the control group will remain relatively stable across all assessments.*

*6. If the interventions differ in their sustained effectiveness, then the Gita and Yoga+Gita groups will have significantly lower DPI-GAD7 scores compared to the Yoga group at 45 days post-intervention.*

*7. Gender may moderate the effect of the interventions on psychological distress.*

*8. Educational attainment may moderate the effect of the interventions on psychological distress.*

*9. If continued practice of the interventions is important for sustained stress reduction, then participants who continue practicing the interventions will have lower GAD7 scores at the delayed post-intervention assessment than those who do not continue.*

**Design Plan**

Study type

*Experiment - Randomized controlled trial.*

Blinding

*Personnel who analyze the data collected from the study will not be aware of the treatment applied to any given group.*

Is there any additional blinding in this study?

*The researchers will be divided into three teams; each team will have no contact with the others once recruitment begins until data analysis is complete. The participant recruitment will be conducted by Team 1. Team 2 will coordinate with external volunteers and ensure that the interventions are administered smoothly; they will also collect the data. Team 3 will analyze the collected data; they will have no contact with the participants.*

Study design

*This study will use a between-subjects design with four levels (Control, Yoga, Gita, Yoga+Gita), incorporating repeated measures of psychological distress at three time points to assess both immediate and long-term effects of the interventions. Randomization will ensure equal distribution across groups, and no counterbalancing is expected to be needed.*

Randomization

*The study will use simple randomization via a chit-pull system at the individual participant level, ensuring equal and unbiased assignment to one of the four study groups. There will be no additional stratifications or blocks. The chits will be placed in a glass bowl, folded, with no visible marks to enable identification of their contents. Each participant will remove one chit from the bowl, and the chit will contain the participant IDs and the group assignments.*

**Sampling Plan**

Existing Data

*None*

Data collection procedures

*Population:* The participants will be healthcare workers (HCWs) employed at Sri Jagannath Heath Care and Research Centre, Dhanbad, Jharkhand, India. They are facing significant psychological stress during this COVID-19 pandemic, making them suitable candidates for the study aimed at assessing interventions for stress reduction.

*Recruitment:* Participants will be recruited by posting a call for participation on the internal email system and notice board of the hospital. This announcement will include all relevant information about the study, such as the nature of the interventions, expected commitment, and the informed consent form. No payments will be provided for participation.

*Inclusion and Exclusion Criteria:*

Inclusion Criteria: Participants will be aged 18 years or over and be currently employed HCWs at the hospital. They will also need to be available for the entire duration of the intervention (1 week) and follow-up (45 days post-intervention).

Exclusion Criteria: Participants with pre-existing diagnosed mental health conditions will be excluded to ensure that the study focuses on measuring the effect of the interventions on psychological distress caused by work-related stress during the pandemic, rather than on pre-existing disorders.

*Grouping:* After the eligible HCWs return the signed informed consent forms, they will be randomized into one of four groups (Control, Yoga, Gita, Yoga+Gita) using a chit-pull system, as previously described, in a 1:1:1:1 ratio.

*Study Timeline:* Recruitment will begin on October 25, 2021, and end on November 5, 2021. The baseline assessment (B-GAD7) will be administered on November 12, 2021. The interventions (Yoga, Gita, Yoga+Gita) will be conducted between November 13, 2021, and November 19, 2021. The immediate post-intervention GAD7 (IPI-GAD7) will be collected on November 20, 2021 (1 day post-intervention). The delayed post-intervention GAD7 (DPI-GAD7) will be administered on January 3, 2022 (45 days post-intervention).

*Intervention Details:* Participants in the intervention groups will attend daily 1-hour sessions for one week. The Yoga group will practice yoga for the entire hour. The Gita group will attend Bhagavad Gita lessons for one hour. The Yoga+Gita group will have 30 minutes of yoga followed by 30 minutes of Bhagavad Gita lessons. The Control group will not receive any intervention.

*Data Collection Process:* Psychological distress will be measured using the Generalized Anxiety Disorder-7 (GAD7) scale at three time points; Baseline (B-GAD7) - Administered before the interventions starts, Immediate Post-Intervention (IPI-GAD7) - Administered 1 day after the last intervention session, and Delayed Post-Intervention (DPI-GAD7) - Administered 45 days after the intervention. All data will be collected anonymously using only the participant identification numbers (PINs) to maintain confidentiality.

*Data Management:* The consent forms and a document linking participants’ names to their PINs will be securely stored and not accessible to the data analysis team or to the intervention coordination and data collection team. The data will include GAD7 scores and demographic information (age, gender, education level).

Sample size

*The sample size will be calculated to detect significant effects with 80% power to detect a minimum clinically important difference of 4 for the GAD7 with a standard deviation of 3.78 at the standard alpha error of 0.05. A dropout rate of 15% will be considered. PASS 2020, v20.0.6 will be used to calculate the sample size.*

Stopping rule

*None*

**Variables**

Manipulated variables

*None*

Measured variables

*The primary outcome variable is psychological distress, measured using the GAD7 scale at baseline, immediately post-intervention, and 45 days post-intervention. The main predictor variable is the intervention type (Control, Yoga, Gita, Yoga+Gita), and demographic covariates (age, gender, education) will also be collected. Continued practice is a follow-up variable to examine its impact on long-term outcomes.*

**Analysis Plan**

Statistical models

*Descriptive Statistics:* Before any inferential tests, we will compute descriptive statistics (mean ± standard deviation for continuous variables and frequencies/percentages for categorical variables) to describe the demographic characteristics (age, gender, education level) and baseline GAD7 scores for each group.

*Tests for Normality:* To determine the appropriate statistical tests, we will first assess the normality of the GAD7 scores using the Shapiro-Wilk test. If the data are normally distributed, we will proceed with parametric tests. If the data do not meet normality assumptions, we will use non-parametric alternatives.

*Primary Plan (Parametric Tests):*

Within-Group Comparisons: To examine changes in GAD7 scores over time (Baseline, Immediate Post-Intervention, and Delayed Post-Intervention) within each group, we will use repeated-measures ANOVA. If significant, we will conduct pairwise t-tests with Bonferroni correction to compare GAD7 scores between each pair of time points (e.g., Baseline vs. IPI-GAD7, Baseline vs. DPI-GAD7).

Across-Group Comparisons: To compare GAD7 scores between the four groups (Control, Yoga, Gita, Yoga+Gita) at each time point (Baseline, IPI-GAD7, DPI-GAD7), we will use one-way ANOVA. If significant differences are found, we will apply Tukey’s HSD for post-hoc pairwise comparisons between groups.

Subgroup Analysis (by Gender, Educational Attainment, and Continued Practice): For subgroup analyses, we will use two-way ANOVA to assess whether gender (male, female), education level (Bachelor’s vs. Master’s or higher), and continued practice (yes, no) influence GAD7 score changes over time or interact with the interventions.

Effect Size Calculations: Partial eta-squared (η²) will be calculated for repeated measures ANOVA and η² will be calculated for one-way ANOVA to estimate the effect sizes.

*Backup Plan (Non-Parametric Tests):*

Within-Group Comparisons: We will use the Friedman Test to compare GAD7 scores over time within each group (Baseline, IPI-GAD7, DPI-GAD7). If significant, we will perform pairwise comparisons using the Conover Test to identify specific differences between time points.

Across-Group Comparisons: To compare GAD7 scores across the four groups at each time point, we will use the Kruskal-Wallis Test. If significant, we will conduct the Dunn’s Test for pairwise group comparisons.

Subgroup Analysis (by Gender, Educational Attainment, and Continued Practice): For the gender, education, and continued practice subgroup analyses, we will use the Mann-Whitney U Test to compare GAD7 scores between subgroups at each time point.

Effect Size Calculations: We will compute the Kendall’s W for effect size in the Friedman test and η² for the Kruskal-Wallis test to estimate the effect sizes. Statistical Significance: For all tests, a two-tailed p-value < 0.05 will be considered statistically significant.

Transformations

*None*

Data exclusion

*Participants who miss any follow-up session (B-GAD7, IPI-GAD7, DPI-GAD7) will be excluded.*

**Other disclosures**

A similar study involving homeless persons is also planned in the future.
