# Supplementary material for: The stress reduction potential of Bhagavad Gita and Yoga for healthcare workers during the COVID-19 pandemic: A randomized controlled trial
Source: PLoS One. 2026 Apr 24;21(4):e0347320. doi: 10.1371/journal.pone.0347320 (PMC13108764; doi:10.1371/journal.pone.0347320)
Supplement: Attachment — Submitted filename: point-by-point responses.docx [file pone.0347320.s004.docx]

Baltimore Tabios Bejosano
*PLOS ONE*

Dear Editor:

Thank you for giving us the opportunity to revise our manuscript to comply with the journal guidelines. We have gone through the comments sent to us and have made the required changes to the manuscript. We have also prepared a point-by-point response to your comments below.

We hope that our manuscript now meets all the requirements of the journal. We look forward to your further feedback and to the review process.

Thank you for your consideration.

Sincerely,

Laalithya Konduru

Point-by-point responses

1. Please complete all items on the Human Participants Research Checklist that are relevant for your submission, by following this link: <http://journals.plos.org/plosone/s/file?id=dc11/PLOSOne_Human_Subjects_Research_Checklist.docx> (Contact us at plosone@plos.org if you cannot access the document.) There may be overlap between the checklist items and other queries listed below; please address any duplicated queries both in your response email and on the checklist itself. Upload the completed Human Participants Research Checklist as file type “Other” when you re-submit your manuscript. This document is for internal journal use only and will not be published if your article is accepted. The requested information will help us to assess whether your submission complies with PLOS ONE’s policies and adheres to applicable reporting standards. Note that your manuscript may be rejected if you provide incomplete or inadequate responses to the checklist questions and that changing the
   ‘Section/Category’ of your article does not affect this requirement.

**RESPONSE: We have submitted the completed checklist, and as required by the checklist, we have uploaded the ethics approval document. Please note that there are two ethics approval documents – the first approval was granted to recruit 42 participants and the second approval was granted to increase the sample size to 68. We have uploaded both documents.**

2. Please provide additional details regarding participant consent. In the ethics statement in the Methods and online submission information, please ensure that you have specified what type you obtained (for instance, written or verbal, and if verbal, how it was documented and witnessed). If your study included minors, state whether you obtained consent from parents or guardians. If the need for consent was waived by the ethics committee, please include this information.

Once you have amended this/these statement(s) in the Methods section of the manuscript, please add the same text to the “Ethics Statement” field of the submission form (via “Edit Submission”).

For additional information about PLOS ONE ethical requirements for human subjects research, please refer to <http://journals.plos.org/plosone/s/submission-guidelines#loc-human-subjects-research>.

**RESPONSE: We have made it clear in the methods that written informed consent was obtained. We have also updated the statement in the submission system.**

3. Please note that you must upload a completed CONSORT flowchart as figure 1 of your manuscript. Blank copies of this document and information regarding CONSORT can be found via the following link: (<https://www.equator-network.org/reporting-guidelines/consort/>).

**RESPONSE: We have included the CONSORT flowchart as Fig 1.**
