# Supplementary material for: The stress reduction potential of Bhagavad Gita and Yoga for healthcare workers during the COVID-19 pandemic: A randomized controlled trial
Source: PLoS One. 2026 Apr 24;21(4):e0347320. doi: 10.1371/journal.pone.0347320 (PMC13108764; doi:10.1371/journal.pone.0347320)
Supplement: Attachment — Submitted filename: COMMENTS.docx [file pone.0347320.s003.docx]

**Strengths of this Manuscript:**

- The study addresses a significant gap by evaluating culturally tailored interventions for HCWs in India, where Western mental health strategies may not be fully applicable.
- The use of a randomized controlled trial design with a control group strengthens the validity of the findings.
- The interventions are well-described, and the use of validated tools (GAD-7) for outcome measurement is appropriate.
- The manuscript highlights the importance of cultural sensitivity in mental health interventions, which is a valuable contribution to the literature.

**Major Comments**

1. **Sample Size and Generalizability:** The study included 68 participants (17 per group), which is relatively small. While a power calculation was performed, the limited sample size and single-center design may restrict the generalizability of the findings. Please discuss this limitation more explicitly in the discussion section.
2. **Randomization and Blinding:** The chit-pull method for randomization is described, but more details on allocation concealment and how potential selection bias was minimized would strengthen the methodology section.

Participants were aware of their group assignments, which could introduce performance bias. While data analysts were blinded, the lack of participant blinding should be acknowledged as a limitation.

3. **Statistical Analysis**

The manuscript mentions the use of non-parametric tests due to non-normal GAD-7 distributions. Please clarify whether adjustments for multiple comparisons were made during subgroup analyses to control for type I error.

It would be helpful to report effect sizes alongside p-values to provide a sense of the practical significance of the findings.

4. **Intervention Fidelity and Adherence**

The study mentions that participants were asked about continued practice at follow-up. Please provide more detail on how adherence to the interventions was monitored and whether this influenced the outcomes.

5. **Control Group Considerations**

The control group received no intervention. Consider discussing whether an attention control or placebo intervention could have helped account for the effects of group participation and attention from facilitators.

**Minor Comments**

- The abstract and introduction could benefit from more precise language regarding the hypotheses and objectives.
- Please clarify the rationale for selecting only the second chapter of the Bhagavad Gita for the intervention.
- Some references in the introduction are cited as numbers (e.g., , ) but the reference list is not included. Ensure all references are properly listed and formatted.
- There are minor typographical and grammatical errors throughout the manuscript that should be addressed during proofreading.

The manuscript presents promising findings on the use of culturally tailored interventions for reducing psychological distress among HCWs during the COVID-19 pandemic. Addressing the points above will strengthen the manuscript and its contribution to the field.
