# Supplementary material for: The stress reduction potential of Bhagavad Gita and Yoga for healthcare workers during the COVID-19 pandemic: A randomized controlled trial
Source: PLoS One. 2026 Apr 24;21(4):e0347320. doi: 10.1371/journal.pone.0347320 (PMC13108764; doi:10.1371/journal.pone.0347320)
Supplement: Attachment — Submitted filename: response to reviewers.docx [file pone.0347320.s005.docx]

Point-by-point responses

### **Reviewer #1**

**C1. Was a t-test used for the sample size calculation?
R1.** Yes, although non-parametric methods were later applied due to observed data non-normality, the a priori power calculation assumed normality and employed a two-sided independent samples t-test model.

Original:

PASS 2020, v20·0·6 (NCSS LLC, UT, USA) was used to calculate the sample size for an α of 5%, 1-β of 80%,...

Revised:

PASS 2020, v20·0·6 (NCSS LLC, UT, USA) was used to calculate the sample size based on a two-sample t-test of mean change in GAD-7 scores for an α of 5%, 1-β of 80%,...

**C2. Consider performing an analysis of the proportion of participants who achieved the minimum clinically important difference of 4 by treatment group.
R2.** Thank you for your suggestion. We have now calculated proportions of participants achieving a ≥4-point reduction in GAD-7 scores from baseline to DPI across groups. These proportions were: Yoga, 17·6%; Gita, 47·1%; Yoga+Gita, 41·2%; Control, 11·8% (See Table 2).

Also, we have calculated MCID for subgroups, see Table 5 for “GAD7 values by gender and group over time”, Table 6 for “GAD7 values by educational level and group over time”, Table 7 for “Impact of continued practice on DPI-GAD7 scores”.

Text added to Methods (section 2.5): For exploratory estimation of clinical effect, odds ratios and 95% confidence intervals were calculated using exact methods for 2×2 contingency tables (conditional maximum likelihood estimation), given the small cell counts, comparing MCID responder status in each intervention arm versus control.

Text added to Results (section 3.3.1): Compared with Control, the odds of achieving MCID were higher in the Gita group (OR=6.67, 95% CI 1.15–38.60) and in the Yoga+Gita group (OR=5.25, 95% CI 0.90–30.62), whereas the Yoga group showed little difference (OR=1.61, 95% CI 0.23–11.09). Confidence intervals were wide due to the small sample size.

Text added to Discussion: In exploratory responder analyses, the odds of achieving a clinically meaningful reduction in GAD-7 (≥4 points) were higher in the Gita arm compared with Control (OR = 6.67, 95% CI 1.15–38.60), with a similarly elevated but imprecise estimate observed in the Yoga+Gita arm (OR = 5.25, 95% CI 0.90–30.62). In contrast, Yoga alone showed little difference relative to Control (OR = 1.61, 95% CI 0.23–11.09). These effect size estimates suggest that inclusion of structured Bhagavad Gita–based cognitive reframing may increase the likelihood of clinically meaningful anxiety reduction among healthcare workers. However, confidence intervals were wide, reflecting limited precision due to the small sample size. The study was not powered for categorical responder outcomes; therefore, these findings should be interpreted as exploratory and hypothesis-generating rather than definitive evidence of superiority. Larger trials powered specifically for categorical responder outcomes are required to confirm these estimates.

**C3. If a nonparametric method was used, it would be better to report the median and IQR instead.**
 **R3.** Thank you for pointing this out. We have revised all tables to report GAD-7 values at each time point as median [IQR], consistent with the nonparametric analyses performed. Mean±SDs have been removed.

**C4. To analyze the longitudinal data, a linear mixed model or GEE approach is recommended. These methods provide more statistical power and are more robust to violations of normality assumptions. Within-group and between-group analyses can be included in one model.**
 **R4.** We appreciate the suggestion to use a linear mixed model or GEE. Because our sample size was calculated for a two-sample t-test and the observed GAD-7 distributions were non-normal, we used Friedman and Kruskal–Wallis tests as the most robust choice for this study. We agree that mixed models offer advantages for larger studies, and we have already powered an ongoing follow-up RCT in a different target population using GEE. We have clarified this in the Discussion as a limitation and future direction.

Added text:

Although linear mixed models or generalized estimating equations would model longitudinal correlation more efficiently, given the small sample size and the multi-arm repeated-measures design, mixed-model or generalized estimating equation estimates—particularly for group-by-time effects—would be imprecise and potentially unstable; we therefore used nonparametric tests given the observed non-normality. To address this limitation, our ongoing larger trial of these interventions in a different target population is powered specifically for mixed-model analysis [31].

Reference #31: Konduru L, Dahia SS, Kothari-Speakman G. Registered report: The effectiveness of a Bhagavad Gita intervention to reduce psychological distress in homeless people-A randomised controlled trial. *Plos ONE* 2024; **19:** e0310795.<https://doi.org/10.1371/journal.pone.0310795>.

**C5. Hypothesis testing is not recommended for subgroup analyses, as these analyses are underpowered for such a small sample size. Non significant results are inconclusive. Even statistically significant findings may be due to chance, and appropriate adjustments for multiple comparisons would be necessary.**
 **R5.** We explicitly state in the Methods, Results, and Discussion that subgroup and continued-practice analyses were exploratory, underpowered, and not adjusted for multiple comparisons; accordingly, they carry an increased risk of type I error and should be interpreted as hypothesis-generating.

**C6. A figure displaying group means over time is recommended.**
 **R6.** Thank you for the suggestion. Because GAD-7 values were non-normally distributed and analyzed using nonparametric methods, we added a figure (Figure 2) plotting median (IQR) GAD-7 scores at baseline, immediately post-intervention, and 45 days post-intervention for each group, consistent with our descriptive and inferential reporting. Panels additionally stratify trajectories by gender and educational level; these subgroup displays are exploratory.

### **Reviewer #2**

#### **2.1. Study Design & Randomization**

#### **1. Randomization Method (Chit-Pull)**

#### **o Issue: Simple randomization in small samples (n = 68) can yield baseline imbalances by chance. Although the authors report no significant differences in age, gender, or education (ANOVA p > 0.7 for all), they did not present the baseline GAD-7 distributions across groups in order to confirm statistical equivalence on the primary outcome variable.**

Response: Thank you for this comment. We now present baseline GAD-7 values (median [IQR]) by group in Table 2 and explicitly report that baseline GAD-7 did not differ significantly across groups (Kruskal–Wallis p = 0.908; Results, section 3.3.1). We have also corrected the baseline demographic comparisons in Results, section 3.2 to reflect the nonparametric (Kruskal–Wallis) and χ² tests described in the Methods.

#### **2. Sample Size Calculation & Power**

#### **o The authors used PASS 2020 to calculate n = 68 (17 per arm) based on: α = 0.05, power = 80%, minimum clinically important difference for GAD-7 of 4, SD = 3.78 (from a previous study), and a 15% dropout.**

#### **o Clarification Needed: It would be helpful to specify whether the sample size calculation was powered to detect differences between each pair of groups or only to detect a difference between “any intervention” versus control. Given four arms, a calculation designed for a two-group comparison may not yield adequate power for pairwise comparisons among three intervention arms.**

#### **o Recommendation: Briefly clarify in Methods whether the calculation aimed for (a) a two-group comparison (e.g., Gita vs. control) or (b) multiple comparisons (e.g., adjusting for 3 pairwise tests). If the latter, describe any adjustment (such as Bonferroni) made or comment on the potential for type II error in some comparisons.**

Response: Thank you for this important clarification request. The a priori sample size calculation in PASS was based on the default two-group intervention-versus-control comparison (two-sided two-sample t-test, α=0.05) and did not incorporate multiplicity adjustment for all pairwise comparisons among the four arms. We have now clarified this explicitly in Methods, section 2.5. Because post hoc pairwise comparisons were Bonferroni-adjusted in the analysis, some contrasts may have reduced statistical power; accordingly, we acknowledge in the Discussion that the study may be underpowered for certain pairwise comparisons and that type II error is possible.

#### **3. Masking / Blinding**

#### **o The trial was open-label for participants (because interventions are behavioral), but the data analysts were blinded. This is acceptable.**

#### **o Recommendation: In the Discussion, acknowledge that open-label assignment may introduce performance bias (e.g., expectation effects), especially in spiritually oriented interventions. If possible, note any steps taken to minimize this (for instance, emphasizing to participants that “all interventions are hypothesized to reduce stress,” whatever the arm).**

Response: We Thank you for raising this important point. We would like to clarify the randomization and allocation process to avoid any ambiguity.

Randomization was based on a computer-generated allocation sequence created in advance by a researcher not involved in recruitment or enrollment. Each assignment (participant identification number and corresponding group allocation) was placed into a sequentially numbered, opaque, sealed envelope. Prior to enrollment, the sealed envelopes were placed in a container. After providing written informed consent, each participant selected one envelope from the container, which was then opened to determine group assignment. This procedure ensured both random sequence generation and allocation concealment at the time of enrollment. We have revised the Methods section to reflect this process more precisely and to avoid the impression of informal or potentially biased allocation.

Specifically, in the Methods section,

*“Upon returning the signed consent form to the research office, the participants were randomized using the chit-pull system into four equal groups in a 1:1:1:1 ratio. Simple randomization was used. The chits, containing the participant identification number (PIN) and the name of the group to which the participant is assigned, were placed in a fishbowl and folded in such a way that no external marks were visible; the participants picked one chit after returning the signed consent forms.”*

has been updated to:

*“Upon returning the signed consent form to the research office, the participants were randomized in a 1:1:1:1 ratio using a computer-generated random sequence. The sequence, containing participant identification numbers (PINs) and corresponding group assignments, was generated in advance by a researcher not involved in recruitment and placed into sequentially numbered, opaque, sealed envelopes that were handled only by research staff uninvolved in enrollment. The sealed envelopes were placed in a container prior to enrolment. After written informed consent was obtained, each participant selected one envelope from the container, which was then opened to reveal their group assignment. This procedure ensured both random sequence generation and allocation concealment at the time of enrollment.”*

With respect to masking, we acknowledge that the trial was necessarily open-label for participants due to the behavioral nature of the interventions. We now explicitly state in the Discussion that open-label assignment may introduce expectancy or performance bias, particularly in spiritually oriented interventions. In the participant information sheets, we had informed the participants that all interventions are expected to reduce stress. We clarify this in the discussion, along with the fact that outcome assessors and data analysts were blinded to allocation.

**2.2. Intervention Details & Adherence**

**1. Yoga Protocol**

**o The manuscript lists specific asanas and pranayama (e.g., Bhadrasana, Shawasana, Nadi Shodhana Pranayama, etc.), delivered by certified teachers.**

**o Minor Point: The description could specify whether each session included a standardized sequence (e.g., warm-up, standing postures, seated postures, pranayama, meditation) and how long each component lasted.**

**o Recommendation: Add a brief sentence (Methods §2.2) confirming that each one-hour session followed a standardized format (e.g., “Each session began with a 5-minute centering meditation, 20 minutes of asana practice, 20 minutes of pranayama, and concluded with 15 minutes of guided meditation”). This will help reproducibility.**
Response: We have clarified in Methods, section 2.2 that each one-hour Yoga session (Yoga group) followed a standardized format (30 min asana, 15 min pranayama, 15 min guided meditation), improving reproducibility. We also mention that the 30-min Yoga sessions (Yoga+Gita group) followed the same sequence, with each component proportionally reduced in duration. We also clarify that the same instructors conducted all Yoga sessions throughout the intervention period.

**2. Bhagavad Gita Protocol**

**o Participants in Gita and Yoga+Gita attended one-hour daily sessions learning and discussing Chapter 2 of the Gita.**

**o Minor Point: It would be useful to know who delivered these sessions (e.g., trained teachers of ISKCON), how they structured learning (reading aloud, discussion, Q&A), and whether participants received any written materials or audio recordings to reinforce learning during follow-up.**

**o Recommendation: Clarify in Methods if handouts/translations were provided, whether the same instructor led each session, and if participants were encouraged to review the verses during the 45-day follow-up period (beyond simply asking whether they “continued practice”).**

Response: Thank you for this helpful suggestion. We have clarified in Methods (section 2.2) that the Bhagavad Gita sessions were delivered by trained teachers from the International Society for Krishna Consciousness (ISKCON), Dhanbad, using the text Bhagavad-Gita As It Is. Sessions consisted of structured verse reading followed by explanation and discussion. Printed handouts of the verses were provided to participants, the same instructor delivered all sessions to ensure consistency, and participants were encouraged to review the verses during the 45-day follow-up period. These additions improve clarity and reproducibility of the intervention.

**3. Measurement of Continued Practice**

**o The authors divided intervention groups into subgroups (“continued practice” vs. “did not continue”) at 45 days. However, Table 7 (p. 28) shows that in the Yoga+Gita group, those who did not continue practice had a lower DPI-GAD7 (2.455 ± 1.753) than those who did continue (4.833 ± 2.317), yet the text (Results §3.4.3) states that continued practice was associated with lower DPI-GAD7 scores.**

**o Major Concern: This apparent discrepancy between text and table must be resolved. It is unclear whether the table cells have been erroneously swapped, whether the text wrongly interprets the table, or whether a coding error occurred.**

**o Recommendation: Please double-check the raw data and clarify which subgroup’s mean is which. If indeed those who continued had lower stress scores, the table’s labels need to be reversed; if not, correct the text to reflect the data. In either case, add a brief explanation (e.g., “Participants who reported continuing the practices for 45 days scored X vs. Y in DPI-GAD7 (p = .032), indicating a sustained benefit among adherents”).**

Response: Thank you for catching this discrepancy. Upon careful review, we confirmed that the discrepancy arose from a labeling error in the earlier version of Table 7, where the “continued practice” and “did not continue” subgroup labels were inadvertently reversed. The table has now been corrected to accurately reflect that participants who continued practice had lower DPI-GAD7 scores than those who did not. We also corrected a minor rounding inconsistency in the Results section; the p-value for the Yoga+Gita group is now consistently reported as p = 0.033 throughout the manuscript.

**2.3. Statistical Analysis**

**1. Choice of Nonparametric Tests**

**o The Shapiro–Wilk test indicated that demographic variables were normally distributed but GAD-7 scores were not, justifying Friedman (within-group) and Kruskal–Wallis (between-group) tests. Pairwise comparisons used Conover (for Friedman) and Dunn’s (for Kruskal–Wallis).**

**o Strength: This is appropriate for small samples with non-normal distributions. The use of effect sizes (η² for between-groups; Kendall’s W for within-groups) is commendable.**

**o Issue: The manuscript does not explicitly state whether p-values were adjusted for multiple pairwise comparisons (for instance, if Dunn’s test used a Bonferroni correction or a false discovery rate). Although many software packages automatically adjust, this should be explicitly noted.**

**o Recommendation: In the Statistical Analysis subsection, specify whether and how Dunn’s and Conover’s tests were adjusted for multiple comparisons. If no adjustment was made, comment on the possibility of inflated type I error rates and whether findings near the 0.05 threshold (e.g., p = 0.047 for Gita vs. control at DPI) remain robust after correction.**
Response: We now explicitly state in Methods (section 2.5) that Dunn’s and Conover–Friedman post-hoc tests were Bonferroni-adjusted. We also clarify that subgroup analyses were exploratory and not multiplicity-adjusted.

**2. Presentation of Effect Sizes**

**o The authors report η² = 0.166 for IPI-GAD7 and η² = 0.140 for DPI-GAD7.**

**o Minor Point: It may be helpful to remind readers of conventional benchmarks (e.g., small = 0.01, medium = 0.06, large = 0.14 for η²; or describe Kendall’s W magnitude).**

**o Recommendation: Briefly state in the Results (e.g., “η² = 0.166 indicates a large effect size according to Cohen’s conventions for nonparametric tests”) to assist interpretation.**

Response: We have added interpretive guidance in the Results, stating that η² ≥ 0.14 represents a large effect (Cohen’s convention), and that Kendall’s W ≈ 0.40 reflects moderate-to-large concordance.

**2.4. Results & Data Presentation**

**1. Baseline Comparisons**

**o Although Table 1 shows no significant baseline differences in demographics, baseline GAD-7 by group is not explicitly compared.**

**o Recommendation (echoing §2.2.1): Add a row in Table 2 or a supplementary table indicating baseline (B-GAD7) differences (median and IQR) with a corresponding Kruskal–Wallis p-value. If not significant, a brief statement in the text reiterating “Baseline GAD-7 did not differ significantly across groups (Kruskal–Wallis p = 0.908)” would suffice.**

Response: Baseline GAD-7 comparisons are now reported in Results (section 3.3.1; Kruskal–Wallis p = 0.908) and shown in Table 2.

**2. Tables & Figures**

**o Table 2 succinctly presents mean ± SD and percent change; however, percent change uses the formula “100×((DPI-GAD7)−(B-GAD7))/(B-GAD7)”. In the equation printed under the table, there is a missing “7” in the denominator (i.e., “(B-GAD−7)” rather than “(B-GAD7)”).**

**o Recommendation: Correct the typographical error in the formula and ensure consistency (e.g., “(B-GAD7)” without stray hyphens).**

Response: The typographical error in the %Change formula has been corrected throughout.

**o Table 7 Labeling Discrepancy (discussed in §2.2.3). Once clarified, ensure that subheadings (Yes vs. No) clearly correspond to “Continued practice” versus “Did not continue.”**
Response: The table has been corrected to ensure that there are no labeling errors.

**o Figure 1 (CONSORT Flowchart) is present and correctly depicts participant flow. Ensure the figure caption briefly states total assessed (n = 79), excluded (n = 11), randomized (n = 68), and final analysis (n = 68), per CONSORT guidelines.**

Response: The CONSORT caption has been revised to include total assessed, excluded, randomized, and analyzed, consistent with CONSORT guidelines.

**3. Subgroup Analyses**

**o The authors present subgroup analyses by gender (Table 5) and education (Table 6). Although interesting, these stratified tests inflate type I error risk.**

**o Recommendation: Acknowledge in the Discussion (Limitations) that subgroup findings (especially gender differences) are exploratory and warrant replication in larger, adequately powered samples. Emphasize that these results should be interpreted with caution.**
Response: We explicitly labeled all subgroup analyses as exploratory in the Results section (Section 3.4) in addition to the limitations in the Discussion section.

**2.5. Discussion & Interpretation**

**1. Integration with Prior Literature**

**o The Discussion situates findings within existing research on RSIs (religious/spiritual interventions) and mindfulness for HCWs (e.g., Chiappetta et al., Hook et al.), which is appropriate.**

**o Strength: The authors clearly articulate how yoga addresses immediate physiological stress (parasympathetic activation) while the Gita fosters cognitive‐emotional shifts, supporting the complementary hypothesis.**

Response: Thank you for your positive assessment.

**2. Generality & Cultural Context**

**o The manuscript acknowledges that participants were almost all practicing Hindus, limiting generalizability to HCWs of other faiths or secular orientations.**

**o Recommendation: Consider adding a sentence on potential cultural transferability—e.g., whether equivalent spiritual texts or meditation practices from other traditions might yield similar benefits. This will help readers in non-Hindu contexts appreciate how the model might be adapted.**

Response: We added a sentence in the Discussion noting that analogous spiritual texts or secular contemplative practices in non-Hindu contexts may yield similar benefits and merit evaluation in future research.

**3. Limitations**

**o The authors list sample size constraints, recruitment bias (self-selection by spiritually inclined HCWs), and lack of religiosity measurement at baseline.**

**o Additional Limitation: Since adherence beyond self-report (“continued practice”) was binary and based on participant recall, there is potential misclassification. Suggest acknowledging that objective measures (e.g., practice logs or digital tracking) would strengthen future trials.**

**o Recommendation: In Limitations, add:**

**“Because ‘continued practice’ was assessed via self-report at 45 days, social desirability or recall bias may have affected subgroup classification. Future RCTs could incorporate practice diaries or digital reminders to more objectively assess long-term adherence.”**

Response: We added an explicit limitation noting that adherence/continued practice was self-reported and may be affected by recall and social desirability bias, and recommended objective monitoring in future studies.

**2.6. Ethical & Reporting Considerations**

**1. Ethics Statement**

**o Ethics approval (SJHRC–NI/21/OCT/07) and written informed consent are clearly stated (Methods §2.2 and Ethics Statement).**

**o Minor Point: Confirm that the Ethics Statement in submission metadata exactly mirrors the Methods section (including the consent type and any waiver if applicable).**

**o Recommendation: In the submission form, ensure “Written informed consent was obtained” appears under “Ethics Statement” verbatim.**
Response: We ensured that both the manuscript Methods section and the online submission form state identically that “written informed consent was obtained from all participants prior to enrollment.”

**2. Data Availability**

**o The authors state that “All relevant data are within the manuscript and its Supporting Information files,” with a Figshare DOI (https://doi.org/10.6084/m9.figshare.27285234) for individual participant data and dictionary, plus OSF registration info. This aligns with PLOS ONE policy.**

**o Recommendation: Consider specifying if the Figshare repository is publicly accessible immediately upon publication or requires any embargo/permissions. If there is no restriction, a statement such as “The Figshare dataset (DOI: …) is publicly accessible without restriction” would be ideal.**
Response: We clarified in the Data Sharing Statement that the Figshare dataset is publicly accessible without restriction.

**3. Reporting Guidelines**

**o The inclusion of a completed CONSORT flowchart (Fig 1) is appropriate. However, the manuscript would benefit from an explicit statement that the reporting adheres to the CONSORT 2010 checklist.**

**o Recommendation: In Methods §2 (or at the end of the Introduction), add:**

**“Reporting of this RCT follows the CONSORT 2010 guidelines (Schulz, Altman, & Moher, 2010). A completed CONSORT checklist is provided as Supporting Information.”**

Response: We added a statement in the Methods section that the trial is reported in accordance with the CONSORT 2010 checklist and that the completed checklist is provided in the Supporting Information.

### **Reviewer #3**

**Strengths of this Manuscript:**

**• The study addresses a significant gap by evaluating culturally tailored interventions for HCWs in India, where Western mental health strategies may not be fully applicable.**

**• The use of a randomized controlled trial design with a control group strengthens the validity of the findings.**

**• The interventions are well-described, and the use of validated tools (GAD-7) for outcome measurement is appropriate.**

**• The manuscript highlights the importance of cultural sensitivity in mental health interventions, which is a valuable contribution to the literature.**

Response: Thank you for your positive assessment of our manuscript and the thorough review.

**Major Comments**

**1. Sample Size and Generalizability: The study included 68 participants (17 per group), which is relatively small. While a power calculation was performed, the limited sample size and single-center design may restrict the generalizability of the findings. Please discuss this limitation more explicitly in the discussion section.**

Response: We strengthened the Discussion to explicitly state that the small, single-center sample limits external generalizability beyond similar secondary-care hospitals and comparable healthcare worker populations.

**2. Randomization and Blinding: The chit-pull method for randomization is described, but more details on allocation concealment and how potential selection bias was minimized would strengthen the methodology section.**

**Participants were aware of their group assignments, which could introduce performance bias. While data analysts were blinded, the lack of participant blinding should be acknowledged as a limitation.**

Response: Response: We clarified in Methods that randomization was based on a computer-generated sequence placed into sequentially numbered, opaque, sealed envelopes prepared by a researcher not involved in recruitment. Participants selected envelopes only after providing written informed consent, ensuring allocation concealment. We also explicitly acknowledge in the Discussion that the open-label design may introduce performance or expectancy bias, while outcome assessors and data analysts were blinded.

**3. Statistical Analysis**

**The manuscript mentions the use of non-parametric tests due to non-normal GAD-7 distributions. Please clarify whether adjustments for multiple comparisons were made during subgroup analyses to control for type I error.**

**It would be helpful to report effect sizes alongside p-values to provide a sense of the practical significance of the findings.**

Response: We clarify in Methods (section 2.5) that the Dunn’s and Conover-Friedman post-hoc tests with Bonferroni corrections were used for the group-wise analysis. We also clarified that subgroup analyses were exploratory and not adjusted for multiple comparisons. We report effect sizes (η² and Kendall’s W) alongside p-values and provide interpretive benchmarks in the Results to facilitate understanding of practical significance.

**4. Intervention Fidelity and Adherence**

**The study mentions that participants were asked about continued practice at follow-up. Please provide more detail on how adherence to the interventions was monitored and whether this influenced the outcomes.**

Response: We clarify in Methods that adherence was assessed via a single self-reported yes/no question at 45 days, without frequency or duration data. In the Discussion, we acknowledge that this coarse measure may limit interpretation of sustained effects and recommend objective adherence tracking in future trials.

**5. Control Group Considerations**

**The control group received no intervention. Consider discussing whether an attention control or placebo intervention could have helped account for the effects of group participation and attention from facilitators.**

Response: We have added a limitation noting that the usual-care control did not account for nonspecific attention effects and that future trials may consider an attention-matched comparator arm.

**Minor Comments**

**• The abstract and introduction could benefit from more precise language regarding the hypotheses and objectives.**

**• Please clarify the rationale for selecting only the second chapter of the Bhagavad Gita for the intervention.**

**• Some references in the introduction are cited as numbers (e.g., , ) but the reference list is not included. Ensure all references are properly listed and formatted.**

**• There are minor typographical and grammatical errors throughout the manuscript that should be addressed during proofreading.**

**The manuscript presents promising findings on the use of culturally tailored interventions for reducing psychological distress among HCWs during the COVID-19 pandemic. Addressing the points above will strengthen the manuscript and its contribution to the field.**

**Response:** - We revised the Abstract and the Introduction to state explicit, testable hypotheses, including our primary expectation that the combined Yoga+Gita intervention would yield the greatest reduction in GAD-7 scores, with Gita having more durable effects than Yoga alone.
- We added a brief justification in the Intervention section explaining why Chapter 2 of the Bhagavad Gita was selected, highlighting its focus on detachment, duty, and mind-control.

- We ensured that the reference list is complete and numbered.

- The manuscript has undergone proofreading and identified errors corrected.
